# Prediction of persistency for day 305 of lactation at the moment of the insemination decision

**DOI:** 10.3389/fvets.2023.1264048

**Published:** 2023-11-16

**Authors:** Yongyan Chen, Wilma Steeneveld, Mirjam Nielen, Miel Hostens

**Affiliations:** Department of Population Health Sciences, Faculty of Veterinary Medicine, Utrecht University, Utrecht, Netherlands

**Keywords:** persistency, dairy, prediction model, milk production, insemination moment

## Abstract

When deciding on the voluntary waiting period of an individual cow, it might be useful to have insight into the persistency for the remainder of that lactation at the moment of the insemination decision, especially for farmers who consider persistency in their reproduction management. Currently, breeding values for persistency are calculated for dairy cows but, to our knowledge, prediction models to accurately predict persistency at different moments of insemination are lacking. This study aimed to predict lactation persistency for DIM 305 at different insemination moments (DIM 50, 75, 100, and 125). Available cow and herd level data from 2005 to 2022 were collected for a total of 20,508 cows from 85 herds located in the Netherlands and Belgium. Lactation curve characteristics were estimated for every daily record using the data up to and including that day. Persistency was defined as the number of days it takes for the milk production to decrease by half during the declining stage of lactation, and calculated from the estimated lactation curve characteristic ‘decay’. Four linear regression models for each of the selected insemination moment were built separately to predict decay at DIM 305 (decay-305). Independent variables included the lactation curve characteristics at the selected insemination moment, daily milk yield, age, calving season, parity group and other herd variables. The average decay-305 of primiparous cows was lower than that of multiparous cows (1.55 *10^−3^ vs. 2.41*10^−3^, equivalent to a persistency of 447 vs. 288 days, respectively). Results showed that our models had limitations in accurately predicting persistency, although predictions improved slightly at later insemination moments, with R^2^ values ranging between 0.27 and 0.41. It can thus be concluded that, based only on cow and herd milk production information, accurate prediction of persistency for DIM 305 is not feasible.

## Introduction

Traditionally, 12 to 13 months has been considered to be the economically optimal calving interval for dairy cows (1, 2). Such a calving interval can maximize milk yield per cow per year, making use of peak production at the beginning of every lactation (3, 4). However, whether this yearly calving interval is the most optimal choice for every cow is now being questioned in the literature. First, cows can suffer from a negative energy balance at early lactation, especially high-producing cows (5, 6). Subsequent conception rates might therefore be low as cows may not have recovered from the metabolic problems caused by this negative energy balance (7, 8). Second, a yearly calving interval can result in cows being dried off with a relatively high milk yield at the end of the lactation. This has been described as a risk factor for poor udder health in subsequent lactations (9, 10). Third, a yearly calving interval might be an indication for more metabolic disease treatments per year (11). More costs (labor, veterinarian and insemination) may then be incurred and the cow’s health, welfare and lifespan may be impaired (12, 13).

Extending lactation has been proposed as a solution to solve the above-mentioned issues. By extending lactation, farmers deliberately delay the first insemination moment. Several advantages of extended lactation have been identified (14–16). Extended lactation could benefit cow health and production efficiency due to fewer transition periods in the lifespan of the cow. Extending the voluntary waiting period (VWP) for some cows has resulted in higher milk yield per day of calving interval (14, 17, 18). In addition, extending the VWP can lower milk yield during the last 6 weeks before dry-off and benefit udder health in the following dry period and the next lactation (10, 15, 18). Other advantages of extending lactation are that it may reduce greenhouse gas emissions per kg of milk produced, increase profitability and improve cow welfare (19–21). However, not all cows are suitable for extended lactation and the optimal VWP may vary per cow. It is therefore important to select the right cow for extended lactation (22, 23).

Maintaining milk production in late lactation is a prerequisite for extended lactation (15, 24, 25). Persistent cows decrease their milk yield at a lower rate after the peak day, resulting in a flatter lactation curve than non-persistent cows. Persistency is one of the factors that affect body condition scores at the end of the lactation, thus avoiding the risk of parturition diseases after the subsequent calving (26, 27). From an economic perspective, extending lactation of persistent cows could increase the net partial cash flow at herd level (4). Extended lactations will be more beneficial, especially in herds with more persistent cows (28). Definitions of lactation persistency differ between previous studies. Persistency was defined as the milk yield difference at selected DIMs or the declining slope of milk yield within selected intervals after peak yield (18, 29, 30). Persistency can also be determined by using lactation curve models which quantify the lactation curve based on all available milk yield data (31, 32). One of the lactation curve characteristics that defines the curve is the decay, a lactation curve characteristic that can easily be transformed into other measures of persistency as the number of days it takes to halve milk production in the declining stage of lactation (32).

When deciding on the VWP of an individual cow, it is useful to be aware of the persistency for the remainder of that lactation, especially for farmers who consider persistency in their reproduction management. Predictions of persistency for the current lactation could thus provide additional information to optimize the VWP. Currently, breeding values for persistency are calculated for dairy cows (33–35) but, to our knowledge, prediction models to accurately predict persistency at the moment of insemination are lacking.

This study aims to determine whether it is possible to predict lactation persistency for DIM 305 at different insemination moments (DIM 50, 75, 100, and 125) based on available cow and herd data (excluding breeding values).

## Materials and methods

### Available data

Daily milk production and cow data were obtained for the years 2005–2022 from the MmmooOgle programme (Puurs, Belgium). Originally, the dataset included 95,529,301 milking robot visit records for 44,540 cows in 91 herds located throughout the Netherlands and Belgium. Milking robot visit records refer to detailed records generated by automated milking robots during milking of cows. All robot visits included general cow information (e.g., birth date, calving date, age in days and parity) and milk yield (kg). The number of lactating cows per herd varied between 26 (1%) and 394 (99%) per year, with a mean of 174 cows.

### Preliminary data editing

**Figure 1:**
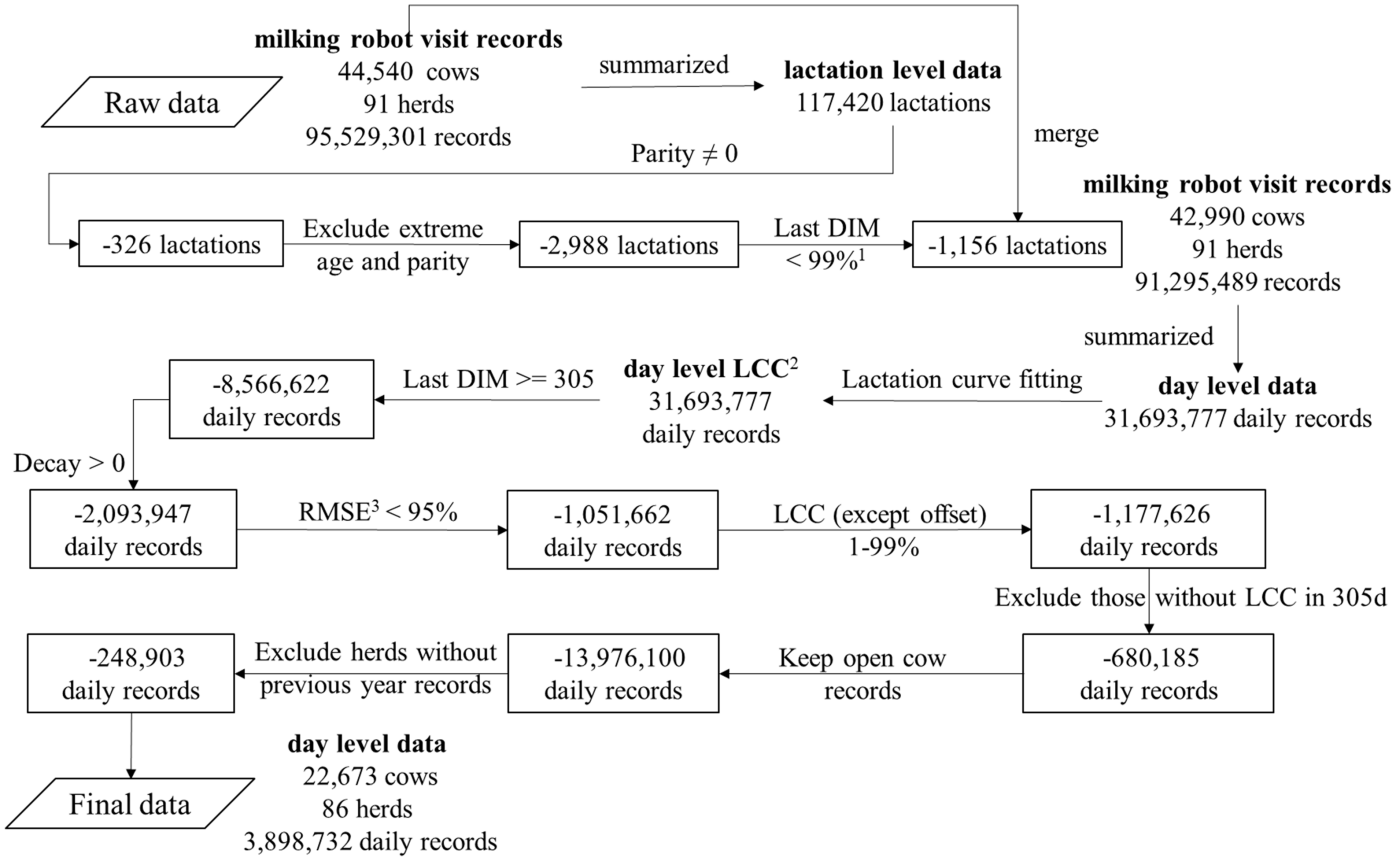
Diagram on data editing of the dataset on milk production per visit to an automatic milking system. The numbers in the boxes represent the excluded numbers. ^1^%, the percentile. ^2^ LCC, lactation curve characteristics (magnitude, time to peak yield, offset and decay). ^3^ RMSE, root mean squared error of the lactation curve fitting.

The data editing diagram is shown in Figure 1. All exact calculations are shown in the Github repository mentioned at the end of this section. First, the milking robot visit records were summarized into 117,420 lactations from 44,540 cows. Subsequently, 326 lactations without parity information were excluded. Percentiles of age in days were calculated within every parity and 2,988 lactations with extreme age in days per parity (>99% percentile or < 1% percentiles) were excluded. In addition, 1,156 lactations with extremely long lactation lengths (>99% percentile) were excluded. Applying these lactation level filters resulted in 91,295,489 milking robot visit records for 42,990 cows in 91 herds. Subsequently, a method (36) from the International Committee for Animal Recording (ICAR) was used to calculate a 24-h milk yield using the 12 previous milkings for every milking robot visit record. The 24-h milk yield of the last milking robot visit record on a given day was considered as the daily milk yield for that specific day. Afterwards, 31,693,777 daily records were summarized in 112,949 lactations. Among these, 34,646 lactations were from primiparous cows while 78,303 lactations were from multiparous cows.

### Lactation curve modeling

A lactation curve was fitted for each daily record using the MilkBot model (32) through the MilkBot lactation API^1^. No records were dropped during the fitting process. The full MilkBot equation is shown as:


(1) $$Y\left(t\right)=a\left(1-\frac{e^{\frac{c-t}{b}}}{2}\right)e^{-dt}$$

where Y(t) is the estimated milk production when DIM is t, and scale a, ramp b, offset c and decay d are the lactation curve characteristics (LCC) describing the lactation curve. LCC are estimated for every daily record by fitting a lactation curve using the data up to and including that day. For example, LCC at DIM 50 are estimated after a lactation curve was fitted for the daily milk records up to and including DIM 50. Based on Bayesian statistics, the specific population mean lactation curve characteristics were used as prior information, and the priors were previously adjusted to the population of Dutch dairy farms (37). The prior was used to a greater extent when the fitted lactation had fewer daily records.

In the current study, the a (scale) was renamed magnitude of milk production (in kg/day) and the b (ramp) was renamed time to peak yield (in days). The d (decay) was transformed into a measure of persistency (in days) using the equation (32):


(2) $$Persistency=\frac{0.693}{d}$$

Persistency refers to the number of days it takes for the daily milk production to decrease by half during the declining stage of lactation. It can be thought of as the “half-life” of milk production. For instance, if a cow has a persistency of 300 days and reaches its peak yield of 40 kg at DIM 100, it means that this cow will attain a milk yield of 20 kg at DIM 400.

The 305-day milk production (M305, in kg) can be estimated using the equation:


(3) $$M305=\frac{\left(a-ae^{-305d}\right)}{d}+\frac{\left(abe^{\frac{c}{b}}\right)\times \left(-1+e^{-305\left(\frac{1}{b}+d\right)}\right)}{2+2bd}$$

### Further data editing

After fitting the lactation curve model, 31,693,777 daily records with LCC from 42,990 cows in 91 herds remained. Daily records with LCC from lactations ending before DIM 305 (*n* = 8,566,622) were excluded because they did not have LCC for DIM 305. Daily records with negative decay (*n* = 2,093,947) and an extremely bad fitting (root mean squared error (RMSE) of lactation curve fitting >95% percentile, *n* = 1,051,662) were also excluded, as were extreme values for magnitude, time to peak yield and decay (>99% percentile or < 1% percentiles, *n* = 1,177,626). In cases where lactations did not have LCC at DIM 305, LCC at DIM 304 was used as a substitute. This was determined based on the 90th percentile of the closest day to DIM 305. Following this, daily records from lactations without LCC at DIM 305 or 304 were excluded (*n* = 680,185). For every lactation, the calculated conception date was calculated by subtracting 282 days (38, 39) from the subsequent calving date. If no subsequent calving date was present, the breeding status was defined as ‘Never’. The breeding status was defined as ‘Bred’ if the calculated conception date was earlier than the date of the daily record; in all other cases the breeding status was defined as ‘Open’. Only daily records with an ‘Open’ breeding status were further included (excluding ‘Bred’ and ‘Never’ daily records, *n* = 13,976,100). To account for the herd effect, we aggregated herd level lactation curve characteristics (HLCC - herd magnitude, herd time to peak yield, herd offset and herd decay) and herd average 305-day milk production (HM305) from the previous year data, following the method described by Chen et al. (37). In short, we aggregated individual lactations to the calendar year in which the lactation ended. Since LCC differ between primiparous cows and multiparous cows (40–42), we divided herd lactations into two parity groups: primiparous cows and multiparous cows. HLCC was then calculated as the mean of the LCC per parity group per herd for each calendar year, while HM305 was calculated as the mean of M305 per herd for each calendar year. Daily records from lactations without HLCC and HM305 from the previous year were excluded (*n* = 248,903). In addition, age in months was calculated from age in days. The calving season was defined based on the calving month (3–5: Spring; 6–8: Summer; 9–11: Autumn; 12–2: Winter) (43, 44). Two parity groups were defined (primiparous cows and multiparous cows). This method resulted in final dataset with 3,898,732 daily records from 43,430 lactations, 22,673 cows and 86 herds.

From the final dataset of daily records with breeding status ‘Open’, we constructed four datasets. The dataset for DIM 50 included daily records at DIM 50 from cows that was not yet conceived at DIM 50. Likewise, datasets were constructed for DIM 75, 100, and 125, which were considered as potential insemination moments. For lactations where LCC was not available on the exact selected insemination moments, we selected the closest day within the 90th percentile of the corresponding DIM (48, 74, 98, 122). After this selection, we have 99,593 daily records from all selected insemination moments from 37,021 lactations, 20,508 cows and 85 herds.

### Model building

**Figure 2:**
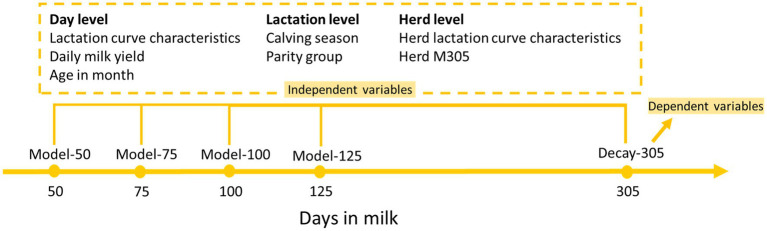
Diagram illustrating the model building procedure.

The model building for each selected insemination moment was carried out separately (Figure 2). In every selected insemination moment, cow-parity records were randomly split into two parts; 80% for the training set and 20% for the test set (Table 1). The training set was used for model training and validation (10-fold cross-validation). The test set was used for model evaluation.

**Table 1:** Number of cow-parity records, cows and herds in training and test set for different selected insemination moments used for model training, validation and evaluation.

Insemination moment (day)	Number of cow-parity records			Number of cows	Number of herds
	Training set	Test set	Total		
50	17,902	4,521	22,423	14,536	83
75	22,006	5,456	27,462	16,764	84
100	19,159	4,752	23,911	15,544	85
125	14,701	3,693	18,394	13,024	84

Due to the right-skewed distribution of persistency and the normal distribution of decay, decay was preferred for statistical analysis and converted to persistency afterwards for a more straightforward interpretation (42). Decay at DIM 305 (decay-305) was therefore defined as the dependent variable. In total, four linear regression models for every selected insemination moment were built to predict decay-305 (Figure 2). The available details at every selected insemination moment were used as independent variables. These included the following cow level variables: LCC, daily milk yield (kg), age in months, calving season and parity group; and herd level variables: HLCC and HM305 from the year preceding the selected insemination moments. HLCC and HM305 were expected to explain herd variance since we could not add herd as the random effect in prediction models. To compare the strength of the effect of each independent variable to the dependent variable, we standardized all continuous independent variables. Funnel graphs were generated to visualize the ranking of the effect size for all continuous independent variables. To validate our method, we also used the same set of data and independent variables to predict M305 and assess the validity of our prediction model approach. The model is shown as:


(4) $$\begin{array}{l} y_{\mathrm{ijkl}}=\mu +LCC_{i}+Dailymilkyield_{i}+Age_{i}+Calvingseason_{j}\\ \;\;\;\;\;\;\;\;\;\;\;\;\;\;+Paritygroup_{k}+HLCC_{l}\;+HM305_{l}+\mu_{\mathrm{ijkl}}\;\;\; \end{array}$$

where y represents the dependent variables (decay-305 or M305), *μ* represents the overall mean, i represents the insemination moments (*i* = DIM 50, 75, 100, or 125), *j* represents the calving season class (*j* = spring, summer, autumn or winter), *k* represents the parity group class (*k* = primiparous cows or multiparous cows), *l* represents the previous year, and $\mu_{\mathrm{ijkl}}$ represents the random residual term from a normal distribution.

### Model evaluation

Model evaluation was carried out on test data with four metrics frequently used in similar research: coefficient of determination (R^2^), RMSE, the mean absolute error (MAE) and the mean absolute percentage error (MAPE) (45–47). R^2^ indicates the proportion of the variance of decay-305 explained by the independent variables. RMSE and MAE indicate the differences between predicted and observed decay-305, with MAE being less sensitive to extreme values in the prediction errors. MAPE measures how much the model’s predictions deviate from the corresponding true value on average, ranging between 0 and 1. We used these four metrics to evaluate all decay prediction models while we only used R^2^ and MAPE to evaluate all M305 prediction models, in order to compare them with the decay models.

Data editing and analysis were carried out using the Python API for the Spark platform (PySpark). Visualization were conducted using GraphPad Prism version 8.0. Code scripts for the data editing steps and statistical analyses can be downloaded at https://github.com/Bovi-analytics/Chen-et-al-2023a.

## Results

Over all lactations the average M305 of primiparous cows (*n* = 11,562) varied between 6,253 (5%) and 11,390 (95%), with a mean of 8,809 kg. The average M305 of multiparous cows (*n* = 15,195) varied between 7,833 (5%) and 13,786 (95%), with a mean of 10,813 kg. The average decay-305 of primiparous cows was lower than that of multiparous cows (1.55 *10^−3^ vs. 2.41*10^−3^, equivalent to a persistency of 447 vs. 288 days, respectively).

Descriptive statistics for the independent variables are only shown for DIM 75 (Table 2); the statistics for the other insemination moments (DIM 50, 100, and 125) can be found in GitHub.

**Table 2:** Descriptive statistics of the dependent and independent variables used in the model predicting decay at DIM 305 (decay-305) at insemination moment DIM 75 based on milk production data from 16,764 cows in 84 Dutch and Belgium herds.

	Primiparous cows				Multiparous cows			
Variables	Mean	SD	5%^a^	95%	Mean	SD	5%	95%
Dependent variable								
Decay-305 (*10^3^, day^−1^)	1.6 ^b^	0.7	0.5	2.9	2.5	0.8	1.2	3.9
Independent variables ^c^								
Cow level variables								
Magnitude (kg)	38.2	6.0	28.2	47.9	51.2	7.5	38.6	63.4
Time to peak yield (day)	28.2	2.4	24.1	31.9	21.0	3.8	13.3	26.4
Offset (day)	−0.50	2.5*10^−5^	−0.50	−0.50	−0.53	0.36	−0.78	0.01
Decay (*10^3^, day^−1^)	1.4 ^b^	0.9	0.2	3.0	1.9 ^b^	1.0	0.3	3.7
Daily milk yield (kg)	32.4	5.6	23.2	41.4	42.7	6.7	31.3	53.6
Age in months	28.2	2.5	25.2	33.1	56.6	18.0	38.0	91.9
Herd level variables								
Herd magnitude (kg)	37.0	3.5	30.9	42.5	49.9	3.8	43.6	56.0
Herd time to peak yield	28.5	1.2	26.7	30.6	21.6	1.3	20.1	23.6
Herd offset (day)	−0.50	1.11*10^−5^	−0.50	−0.50	−0.54	0.09	−0.69	−0.40
Herd decay (*10^3^, day^−1^)	1.5 ^b^	0.4	0.9	2.1	2.2 ^b^	0.3	1.7	2.7
Herd M305 (kg)	9,888	1,001	8,304	11,397	10,002	937	8,449	11,409

^a^ 5 and 95%: the 5 and 95% percentile.

^b^ Persistency was calculated based on the aforementioned decay using the equation 0.693/decay (32). Persistency was not used in the prediction model because of non-normality; decay was used instead. A decay of 1.4, 1.5, 1.6, 1.9, 2.2, and 2.5 *10^−3^ is equivalent to a persistency of 495, 462, 433, 365, 315, and 277 days, respectively.

^c^ All values for the independent variables represent the value at DIM75. Herd variables were aggregated from the day level data of the previous year following the method described by Chen et al. (48).

The model performance indicators of the prediction models for decay-305 at all selected insemination moments are summarized in Table 3A. Among all models, we found higher R^2^ and lower RMSE, MAE and MAPE at later insemination moments. The R^2^ of models for decay-305 range from 0.266 to 0.407, while RMSE, MAE and MAPE were slightly improved along the selected insemination moments.

**Table 3A:** Model performance indicators^1^ of prediction models on test set for decay at DIM 305 at different selected insemination moments (DIM 50, 75, 100, and 125).

Insemination moment (day)	R^2^	RMSE	MAE	MAPE
50	0.266	7.73*10^−4^	6.16*10^−4^	0.391
75	0.270	7.51*10^−4^	5.98*10^−4^	0.400
100	0.325	7.22*10^−4^	5.72*10^−4^	0.371
125	0.407	6.60*10^−4^	5.22*10^−4^	0.370

^1^ Model performance indicators: R^2^, coefficient of determination; RMSE, root mean squared error; MAE, mean absolute error; MAPE, mean absolute percentage error.

**Figure 3:**
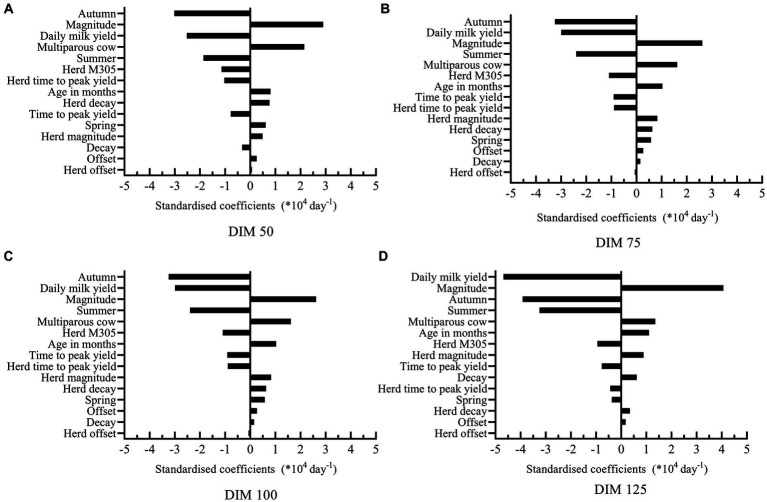
Standardized coefficients of the independent variables used to predict decay at DIM 305 at all potential insemination moments [DIM 50 (A), 75 (B), 100 (C), and 125 (D)]..

Standardized coefficients of the model predicting decay-305 at all potential insemination moments are shown in Figure 3. Among all potential insemination moments, all variables had similar effects on the models. The three most influential variables affecting decay-305 were calving in autumn, daily milk yield and magnitude. However, the specific order of these variables varied across different models. Take model at DIM 75 for example, cows calving in autumn had on average 3.24 (SE = 0.14) lower decay (*10^4^) respectively than calving in winter. Increasing one unit of daily milk yield (7.90 kg) corresponded to an average 2.99 (SE = 0.17) decrease in decay (*10^4^). Increasing one unit of magnitude (9.22 kg/day) corresponded to an average 2.62 (SE = 0.18) increase in decay (*10^4^). Table 3B shows the results of the prediction models for M305, which showed much higher R^2^.

**Table 3B:** Model performance indicators^1^ of prediction models on test set for M305 at different selected insemination moments (DIM 50, 75, 100, and 125).

Insemination moment (day)	R^2^	MAPE
50	0.785	0.073
75	0.850	0.061
100	0.889	0.051
125	0.921	0.043

^1^ Model performance indicators: R^2^, coefficient of determination; MAPE, mean absolute percentage error.

## Discussion

This study aimed to predict lactation persistency for DIM 305 at insemination moments DIM 50, 75, 100, and 125. Our models have low prediction accuracy, although predictions improved at later insemination moments.

In our study, we used decay to measure persistency. The R^2^ of all decay models was under 0.407, suggesting the bad predictive power of the model with all available information included (49). Using the same methodology, we were able to predict M305 much more accurately, thus confirming our prediction methodology to be valid for M305. Similar to previous studies (46, 47, 50), M305 was predictable at all insemination moments with R^2^ values ranging between 0.79 and 0.92 for the different insemination moments. Other methodological approaches were explored to improve the prediction performance of the decay-305 models. First, we explored building prediction models for two parity groups separately. The results were similar (results shown in GitHub). Next to the linear regression, we built models using random forest, lasso regression and ridge regression but results were similar (results shown in GitHub). Models from lasso regression and ridge regression showed the same results to linear regression, indicating that penalization did not improve our models. In addition, adding LCC from the previous lactation did not improve the models in our study (results shown in GitHub).

In the current study, we only included cow and herd information in the prediction models that was available through the MmmooOgle herd management software. As persistency is a heritable trait and could be a target for selection (33, 51, 52) others have tried adding its breeding value to prediction models, though with little success (53). It’s worth noting that the heritability of persistency varies, influenced by factors like the definition of persistency, the breed, and the parity of the cows, with heritability values spanning the range of 0.01–0.33 (34, 52, 54). Breeding values were not available in our dataset. Persistency is furthermore influenced by feed management in herd (55, 56). We took into account this herd-level factor by including HLCC and HM305 into all of our models, rather than including herd as a random effect. This approach allows us to apply our prediction model to unknown farms and effectively consider the impact of herd-level factors on the study outcomes.

There is little existing literature to predict persistency for the mid-late lactation based on data from the beginning of lactation and herd information. We chose to predict persistency for DIM 305 because this is a classic time point for measurements like M305. Other studies chose to predict different parameters to help make insemination decisions. For example, Kjeldsen et al. predicted energy-corrected milk per day of calving interval at DIM 40 for primiparous and multiparous cows separately (53). They included the calving interval in the model while the future calving interval is actually unknown at the moment of making the insemination decision. We assumed that, in their research, predicting milk yield per day of calving interval was equivalent to predicting the milk yield. Another example, Manca et al. (57) used the threshold of daily milk yield at DIM 305 to determine whether a cow is persistent, and defining persistent cows as those with a daily milk yield at DIM 305 greater than 20 kg. Essentially, they used the lactation curve characteristics of the first DIM 90, 120, and 150 to predict the future daily milk yield at DIM 305. The results of Manca et al. (57) correspond with our results on predicting M305 as both achieved a high accuracy. It is important to note that persistency in our study primarily focuses on the slope or rate of decline in milk production over time. Consequently, persistency cannot be directly translated into the exact amount of milk that drops per day without knowledge of the initial peak milk production level. This consideration should be kept in mind when interpreting the findings and conclusions of this study.

Our prediction models could predict M305 well but could not predict persistency for DIM 305 accurately. We hypothesized that M305 is highly predictable due to its association with peak yield (42, 58). Peak yield estimation was commonly established at our insemination moments from DIM 50 onwards (59, 60). In contrast, persistency was not highly correlated with information in early lactation. Additionally, the low prediction accuracy observed in our study may be attributed to other factors that influence persistency between the insemination moments and DIM 305. One potential factor that could impact persistency is pregnancy. However, we were unable to account for the pregnancy effect in our prediction model due to several reasons. Firstly, the exact timing of pregnancy is unknown at the time of making predictions for open cows. Secondly, the quantification of the pregnancy effect on persistency is lacking in previous studies, making it difficult to incorporate it into the model. As a result, we were unable to correct for the pregnancy effect in our prediction model.

There are multiple measures of persistency, and all these measures require the transformation from raw milk data (18, 61, 62). Simple measures of persistency are typically fixed at two time points in lactation (61–63), limiting the ability to observe persistency changes throughout the lactation. To overcome this limitation, we employed lactation curve modeling using the MilkBot model, which allowed to assess persistency at any timepoint within the lactation period. This so called continuous measurement provides insights into the changes of persistency during lactation.

Our data were obtained from AMS farms and we therefore had access to milk production data for each robot visit. Such detailed data did not, however, result in high prediction values. The average M305 of the involved farms was higher than that of average dairy farms in the Netherlands and Belgium (64, 65). Higher milk production can be explained by more frequent milking on AMS farms than on conventional farms (66, 67). In our study, we deliberately only included cows with an ‘Open’ breeding status at the selected insemination moments. ‘Open’ was defined as cows which were not pregnant at the insemination moment but which could be pregnant in the future. Those open cows were the target object of our study since their insemination decisions were yet to be made. In our study, we only included lactations over 305 days, a period commonly accepted by the global standard for livestock data (68).

## Conclusion

Our results showed that based only on cow and herd milk production information, predicting persistency for DIM 305 at different insemination moments (DIM 50, 75, 100, and 125) is challenging. The accuracy of the predictions was found to be low in our models. In order to target decision-support at the insemination moment, other information is needed to improve the accuracy in predicting persistency.

## Data availability statement

The original contributions presented in the study are included in the article/Supplementary material, further inquiries can be directed to the corresponding author.

## Ethics statement

Ethical approval was not required for the studies involving animals in accordance with the local legislation and institutional requirements because the research only involved observational data, without any direct intervention or harm to the animals involved. Written informed consent was not obtained from the owners for the participation of their animals in this study because the research relied on publicly available data from MmmooOgle programme (Puurs, Belgium) and did not involve any direct interactions with the animals or their owners, thereby not requiring explicit consent.

## Author contributions

YC: Conceptualization, Formal analysis, Investigation, Methodology, Software, Validation, Visualization, Writing – original draft, Writing – review & editing. WS: Conceptualization, Methodology, Supervision, Writing – review & editing. MN: Conceptualization, Methodology, Supervision, Writing – review & editing. MH: Conceptualization, Methodology, Supervision, Writing – review & editing.

## Footnotes

^1^ https://api.milkbot.com/
