# Supplementary material for: Prediction of persistency for day 305 of lactation at the moment of the insemination decision
Source: Front Vet Sci. 2023 Nov 16;10:1264048. doi: 10.3389/fvets.2023.1264048 (PMC10687408; doi:10.3389/fvets.2023.1264048)
Supplement: Supplementary file 1 [file Data_Sheet_1.docx]

Supplementary Material


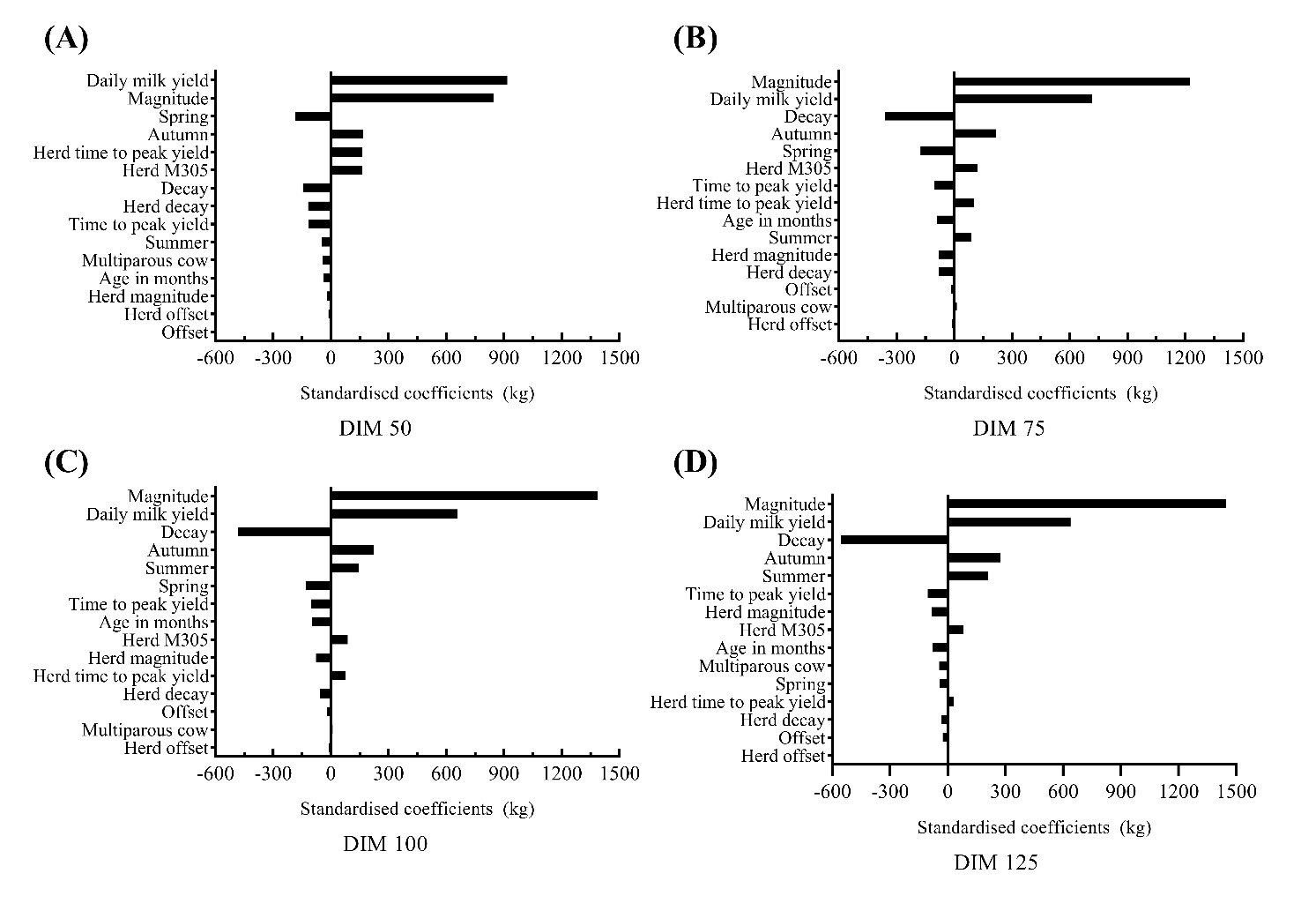


**Supplementary Figure 1.** Standardized coefficients of the independent variables used to predict M305 at all potential insemination moments (DIM 50, 75, 100 and 125).
